# A prognostic fibroblast-related risk signature in colorectal cancer

**DOI:** 10.18632/aging.203677

**Published:** 2021-11-04

**Authors:** Hao Xu, Yisheng Pan

**Affiliations:** 1 Division of General Surgery, Peking University First Hospital, Peking University, Beijing 100034, People’s Republic of China; 2 Translational Cancer Research Center, Peking University First Hospital, Peking University, Beijing 100034, People’s Republic of China

**Keywords:** colorectal cancer, TCGA, GEO, fibroblast, risk signature

## Abstract

Colorectal cancer (CRC) is the third most common cancer in the world. The accessibility of the Cancer Genome Atlas (TCGA) and Gene Expression Omnibus data allows the prognostic evaluation of CRC. Fibroblasts play a key role in the development and progression of tumors while fibroblast-related risk signature in CRC patients has rarely been mentioned. In this study, TCGA data was classified into high-fibroblast and low-fibroblast groups according to the median of fibroblast content. Among 3845 differentially expressed genes between two groups, 14 prognostic genes commonly expressed in GSE39582 and TCGA were identified by LASSO-COX analysis. Then we established a fibroblast-related risk signature in TCGA training group and validated in the GSE39582 testing group. The risk score was significantly associated with the overall survival (OS), and the poor prognosis of patients in high-risk group might relate to the immune cell infiltration in the tumor microenvironment, epithelial-mesenchymal transition, and extracellular matrix related processes. Overall, we proved that the fibroblast-related signature could predict the prognosis of patients which might shed light on the treatment of CRC.

## INTRODUCTION

According to the latest data from 2020, colorectal cancer (CRC) is the third most common and the third most fatal cancer in the United States. The aging population and the prevalence of low-fiber diets will likely lead to a gradual increase in the incidence of CRC. And the 5-year relative survival rate for CRC is 65% which is expected to improve [1]. Therefore, an effective prognostic model is particularly important for the treatment of CRC.

The fibroblast maintains the structural integrity of tissues by synthesizing the extracellular matrix (ECM) of connective tissue [2]. Recent studies revealed the functional heterogeneity of the fibroblast according to the organ of origin and body site, and advances in tumor microenvironment (TME) research also demonstrated fibroblasts played an important role in the progression of cancer [3–6]. Within the TME, cancer-associated fibroblasts (CAFs) had been proved to secrete growth factors, inflammatory ligands, and extracellular matrix proteins which could promote cancer cell proliferation, therapy resistance, and immune exclusion [7]. Single-cell multi-omics sequencing identified fibroblast-specific biomarkers significantly related to the poor prognosis of CRC patients [8]. Although various prognostic models of CRC, such as the hypoxia-related signature, autophagy score signature, and aging-related signature had been developed [9–11], the fibroblast-related risk signature based on open database had rarely been mentioned.

In this study, according to the median of fibroblast content, TCGA data was divided into high- and low-fibroblast groups, the OS was significantly longer in the low-fibroblast group than in the high-fibroblast group. 3845 differentially expressed genes (DEGs) were further identified. Among the 1720 genes shared by TCGA and GSE39582, we finally identified 222 prognostic-related genes by Univariate Cox. Next, based on the LASSO regression analysis, a 14 gene-fibroblast-related risk signature was established, which was significantly associated with the overall survival (OS) of CRC patients in the training and testing groups (Supplementary Figure 1). Besides, the signature was also an independent prognostic factor, whose accuracy was demonstrated using the receiver operating characteristic (ROC) curve. Then, we performed correlation analysis of tumor infiltrating immune cells, protein-protein interactions (PPI), and copy number alteration (CNA) analysis. The gene set enrichment analysis (GSEA) and functional enrichment analysis suggested that epithelial-mesenchymal transition (EMT), and ECM related processes were enriched in high-risk group. Taken together, above results indicated that the fibroblast-related risk signature could predict the OS of CRC patients and the 14 hub genes might be potential therapeutic targets.

## MATERIALS AND METHODS

### Training set collection

Processed RNA-seq data of TCGA CRC and paired clinical information (version: September 8, 2017) were obtained from UCSC Xena webserver (https://xenabrowser.net/). After data quality control (QC) with excluding missing values, a total of 55 normal samples and 375 tumor samples data set were downloaded, and 375 tumor samples data set were collected as training set in this study. The log2 (Counts + 1) value and log2 (FPKM +1) value were both downloaded, if not explained, log2 (FPKM + 1) value means the gene expression in this study.

### Testing set collection

Processed microarray data of CRC and related meta-data were downloaded from NCBI Gene Expression Omnibus (GEO, https://www.ncbi.nlm.nih.gov/geo) with accession code GSE39582. By performing the same QC as training set, we have a validation set with 566 samples.

### Quantification of tumor immune and stromal content

MCP-counter is a wide used computational tool for quantification of tumor immune and stroma content from bulk RNA-seq data [12], such as analysis of immune cells associated with immunotherapy response [13], identification of different TME subtypes [14]. For the ten major cell types in TME, MCP-counter can efficiently derive the semi-quantitative scores which predict the enrichment of specific cell types in a sample based on well-defined marker genes [15]. Thus, we could obtain the quantification value of a specific cellular content and perform inter-sample comparisons in the next analysis.

**Figure 1:**
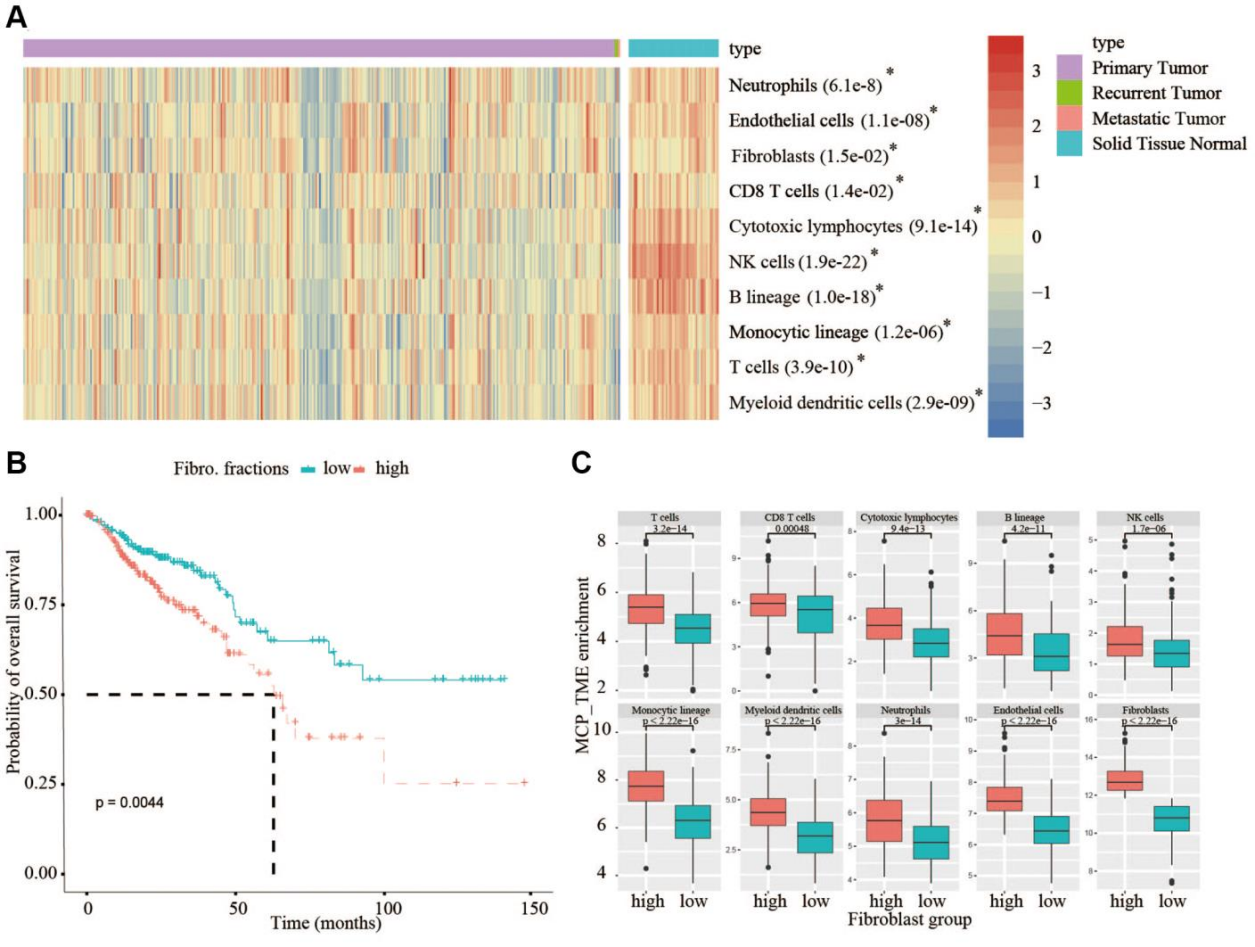
**Analysis of TME in TCGA database and analysis of OS in high- and low-fibroblast groups.** (**A**) In TCGA, all types of immune cells and mesenchymal cells in adjacent normal tissues are higher than that in tumor tissues. (**B**) We divided the 375 patients into a high-fibroblast group and a low-fibroblast group according to the median of the fibroblast content, the OS in the high-fibroblast group is shorter than in the low-fibroblast group. (**C**) The abundance of immune cells in the high-fibroblast group was significantly higher than that in the low-fibroblast group.

In this study, we applied R package MCP-counter (https://github.com/ebecht/MCPcounter) to obtain the abundance scores of ten major stromal and immune cell types (endothelial cells, fibroblasts, CD3+ T cells, CD8+ T cells, cytotoxic lymphocytes, natural killer cells, B lymphocytes, monocytic lineage cells, myeloid dendritic cells and neutrophils). And the enrichment scores of TME were hierarchically clustered by R package pheatmap (Figure 1A) and grouped by the fibroblast enrichment (Figure 1B). Also, after the construction of risk model, we compared the quantification of different cell types across samples grouped into high risk and low risk. Another deconvolution-based software EPIC was also applied for the cellular composition analysis as previously described [16], and the implementation of MCP-counter was documented in detail elsewhere [12].

### Differential expression analysis

For differentiated expressed genes (DEGs) of TCGA with high/low fraction of fibroblasts or high/low risk fibroblast score, we used raw count value of genes as input with R package DESeq2 [17]. Briefly, we performed deseq() function with default parameters, then we defined the DEGs which had higher absolute log2 (fold-change) value than 1.5 and tested significantly (*p*.adj value < 0.05). Out of 3845 DEGs, we chose 1720 genes which had expression in validation set as well. The same criteria was applied to DEG of high/low risk samples.

### Development and validation of the prognostic signature

The OS was defined as the time from diagnosis to death or the last follow-up date. Univariate Cox was used to obtain 222 prognostic genes in training set through traversing all DEGs in fibroblast high/low condition, and LASSO-penalized Cox regression was used to construct optimal prognostic risk signatures in training group. The COX regression model with the LASSO penalty successfully achieved compression and selected 14 fibroblast-related genes simultaneously. The risk score formula was as follows: $\text{risk score = }\sum_{i=1}^{n}\exp i\beta i$ where exp represented the gene expression value while β represented the LASSO coefficient. This model was utilized to estimate the OS of each patient in the training dataset and testing dataset. The predictive potential of the signature was evaluated via area under the curve (AUC) value of the receiver operating characteristic (ROC) curve. The performance of proposed risk score was also tested by calibration curve and DCA curve in both datasets, which were implemented by R package rms and R package rmda, respectively.

### Survival analysis

R package survival and survminer were applied to investigate the difference of prognosis between two groups by log-rank test. By using median expression as a cutoff, we obtained 222 prognostic genes in training set through traversing all DEGs in fibroblast high/low condition. As for prognosis analysis of high risk/low risk in training set, validation set or subgroup analysis of two datasets, we set the median risk score as a metric of risk.

### Enrichment analysis

For pathway enrichment and functional annotation, R packages clusterprofiler and GSEA software were performed [18, 19]. Basically, we investigated upregulated genes (or downregulated genes) whether significantly enriched in particular pathways or terms refer to GO (http://geneontology.org) database. Next, we used a ranked genelist to GSEA algorithm with default parameters, in order to find out which significant pathway (*q* < 0.25) involved the specific condition (i.e., high risk versus low risk) refer to HALLMARK and C2 from MsigDB database (https://www.gsea-msigdb.org/gsea/msigdb).

### Genomic alteration analysis

The related public mutation and copy number alteration data were surveyed and analyzed in online webserver cBioPortal (https://www.cbioportal.org/) [20]. All analysis and plot were followed as the webpage UI guide. Network of gene interactions was obtained from GeneMANIA (https://genemania.org).

### Statistical analysis

All statistical analyses in this study were performed in R version 4.04. *P* < 0.05 was considered as statistical significance. Significance in comparisons of gene expression and putative microenvironment components in figures was calculated by the Wilcoxon Rank-sum test. ROC curves were plotted by R package ROCR. Spearman correlation was the metrics between fibroblast contents and modeled risk scores.

### Data availability statement

All data for this study are available from the corresponding public database.

## RESULTS

### Overall survival correlation

The quantification of tumor stroma is critical to unveil the multi-faceted role of the TME, which may be involved in affecting overall survival (OS) of CRC patients. From TCGA bulk transcriptomic data, we compared the quantification of cellular components of immune cells and mesenchymal cells between 375 cancerous tissues and 55 adjacent normal tissues using the MCP-counter, a tool to estimate cellular content by scoring marker gene expression, the result showed that the content of all types of immune cells and mesenchymal cells in normal tissues are higher than that in cancerous tissues (Figure 1A). Therefore, the TME must play a key role in the occurrence and development of tumors. In view of the crucial roles of the fibroblasts in tumor progression, then we divided the 375 patients into a high-fibroblast group and a low-fibroblast group according to the median of the fibroblast content. From analyzing the relationship between OS and the content of fibroblasts in CRC patients, we observed OS was significantly longer in the low-fibroblast group than in the high-fibroblast group (Figure 1B). Because the TME has an important impact on patients with colorectal cancer, we further investigated the abundance of tumor infiltrating lymphocytes between low- and high-fibroblast groups. We found the abundance of immune cells in the high-fibroblast group was significantly higher than that in low-fibroblast group (Figure 1C).

### Development of a fibroblast-related risk signature

**Figure 2:**
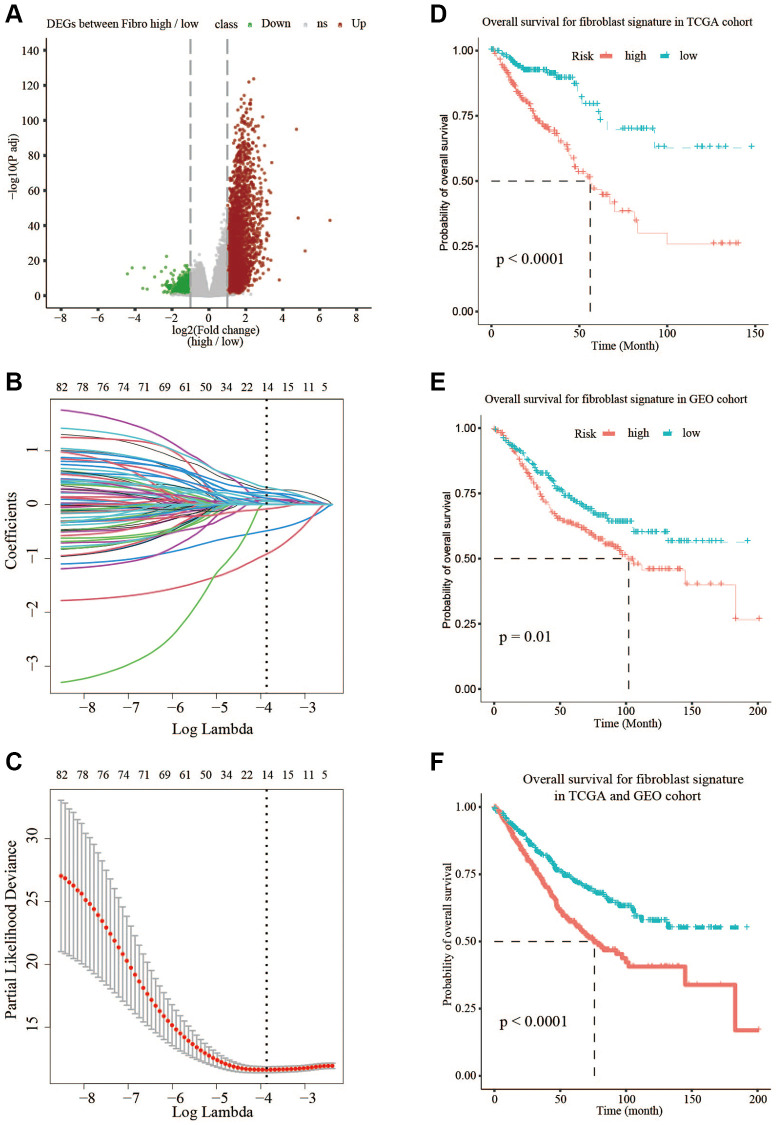
**Prognostic signature based on 14 hub genes.** (**A**) Differentially expressed genes in TCGA between low-fibroblast group and high-fibroblast group. (**B**, **C**) LASSO-COX analysis of prognostic genes. (**D**) The OS is shorter in the high-risk group than in the low-risk group in TCGA. (**E**) The OS is shorter in the high-risk group than in the low-risk group in GSE39582. (**F**) The OS is shorter in the high-risk group than in the low-risk group in TCGA and GSE39582.

To further explore the differences between the high- and low-fibroblast groups, 3845 differentially expressed genes (DEGs) between the two groups were identified in TCGA (Figure 2A). Then, univariate COX analysis was used to identify 222 prognostic genes commonly expressed in TCGA and GSE39582, including 216 risky genes and 6 protective genes. The change in trajectory of each variable was plotted (Figure 2B). We utilized 10-fold cross-validation to construct the model, and showed the confidence interval under each lambda (Figure 2C). When lambda equaled 0.02296418, the model reached the optimal value, and 14 variables were selected (Table 1). Then 14 genes identified by LASSO-COX analysis were used to establish a fibroblast-related risk signature of CRC in TCGA. The formula of the fibroblast-related risk signature was as follows: Risk score = ∑ (βn × expression of gene n). Using the median risk score as a cut-off, CRC patients were divided into low-risk group and high-risk group in TCGA, the OS of the low-risk group was significantly longer than that of the high-risk group (*P* < 0.05) (Figure 2D). To demonstrate the universality of the fibroblast-related signature, the GSE39582 cohort was used to validate the signature. The results confirmed that the fibroblast-related risk signature was significantly associated with the OS of CRC patients in the testing group (Figure 2E). And the analyses of the two databases also demonstrated the validity of the signature (Figure 2F).

**Table 1:** Full names and expression of 14 genes.

**Full names of 14 genes**	**expression**
***RCN3***: Reticulocalbin 3	0.2798524
***RETNLB***: Resistin like beta	–0.4867349
***MMP19***: Matrix metallopeptidase 19	0.0587669
***DACT1***: Dishevelled-binding antagonist of beta-catenin 1	0.2137264
***OLFM2***: Olfactomedin 2	0.3216534
***SCG2***: Secretogranin II	0.0867799
***TUBB6***: Tubulin, beta 6 class V	0.1622284
***REG4***: Regenerating islet-derived family, member 4	–0.0802369
***SLC11A1***: Solute carrier family 11 member 1	0.1206758
***SNCG***: Synuclein, gamma	0.228322
***TREM2***: Triggering receptor expressed on myeloid cells 2	0.080146
***C2orf74***: Chromosome 2 open reading frame 74	0.1174957
***CCL22***: Chemokine (C-C motif) ligand 22	–0.9191942
***CHST3***: Carbohydrate (chondroitin 6) sulfotransferase 3	0.0118777

### Stratified survival assays

**Figure 3:**
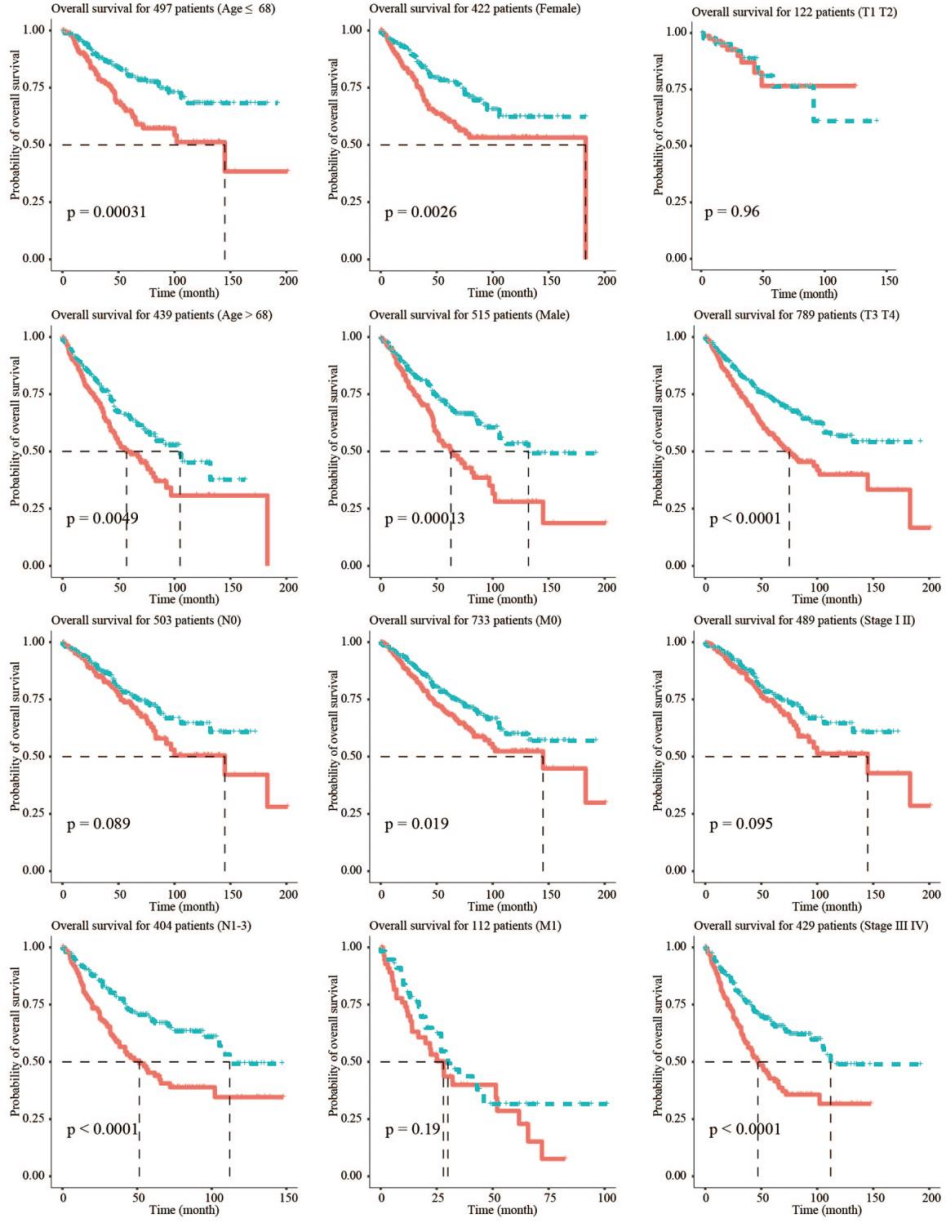
**Stratified survival analysis adjusted to age, gender, stage, and TNM stage.** All CRC patients in the training and testing groups were summarized in the stratified survival analysis. 68 years old was the median age of 937 CRC patients.

To verify the stability of the model and its ability to predict survival in different clinical subgroups, the fibroblast-related risk signature was used to perform stratified survival analysis of CRC patients with survival information in the training and testing groups after adjusting for age, gender, stage, and TNM stage. The clinical characteristics of the training and testing groups were also shown (Table 2). Patients in the different subgroups with missing grouping information were excluded. The results showed that the risk signature could predict the prognosis of CRC patients in most cases, and its prognostic stability was superior in patients with advanced non-metastatic cancer (Figure 3).

**Table 2:** Clinical characteristic of CRC patients in the TCGA and GSE39582.

**Clinical characteristic**	**TCGA (%)**	**GSE39582 (%)**
Total	375	566
Age		
≤68	216 (57.6%)	282 (49.8%)
>68	159 (42.4%)	283 (50%)
Unknown	0	1 (0.2%)
Gender		
Male	206 (54.9%)	310 (54.8%)
Female	169 (45.1%)	256 (45.2%)
T		
T1-T2	66 (17.6%)	56 (9.9%)
T3-T4	306 (81.6%)	486 (85.9%)
Unknown	3 (0.8%)	24 (4.2%)
M		
M0	254 (67.7%)	482 (85.2%)
M1	51 (13.6%)	61 (10.8%)
Unknown	70 (18.7%)	23 (4%)
N		
N0	204 (54.4%)	302 (53.4%)
N1-N3	167 (44.5%)	238 (42.0%)
Unknown	4 (1.1%)	26 (4.6%)
Stage		
I-II	191 (50.9%)	297 (52.5%)
III-IV	165 (44%)	265 (46.8%)
Unknown	19 (5.1%)	4 (0.7%)
Survive		
Yes	289 (77.1%)	371 (65.5%)
NO	86 (22.9%)	191 (33.8%)
Unknown	0	4 (0.7%)

68 years old is the median age of 1,015 CRC patients in our study.

### Validation of the fibroblast-related risk signature

**Figure 4:**
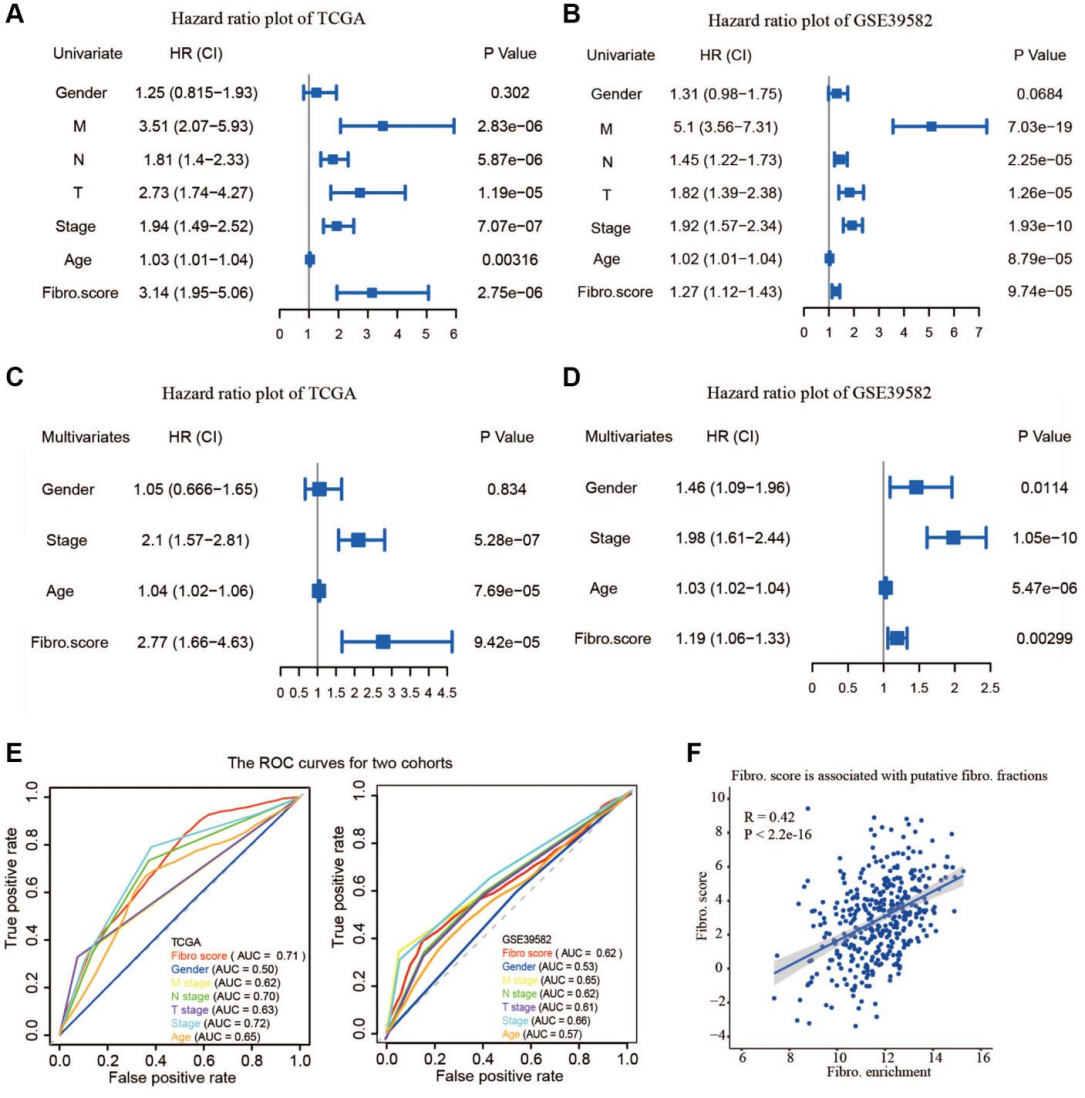
**The validity of prognostic signature and the relationship between the risk score and fibroblast content.** (**A**) Univariate COX regression analysis in TCGA. (**B**) Univariate COX regression analysis in GSE39582. (**C**) Multivariate COX regression analysis in TCGA. (**D**) Multivariate COX regression analysis in GSE39582. (**E**) The receiver operating characteristic (ROC) curve and the areas under the curve verified the accuracy of prognostic signature in the training and testing groups. (**F**) The risk score is associated with the fibroblast content.

To determine whether the fibroblast-related risk signature was a clinically independent prognostic factor, we performed univariate and multivariate analyses and found that age, stage, and risk score were independent prognostic factors in the training group (Figure 4A and 4C). Similar analyses in the testing group GSE39582 led to the same conclusion (Figure 4B and 4D). The results demonstrated that the fibroblast-related signature could be used to predict the survival of CRC patients.

To evaluate the accuracy of this signature, a Receiver Operating Characteristics (ROC) curve was plotted and the area under the curve (AUC) was calculated. Compared to other existing clinicopathological factors, the results showed that the signature is an effective index to predict the OS of CRC patients in both the training group and the testing group (Figure 4E). Moreover, calibration plots indicated that in comparison with an ideal model, the signature had a similar performance (Supplementary Figure 2A). The results of DCA also demonstrated that our signature had high potential for clinical utility (Supplementary Figure 2B). A higher risk score was related to a higher content of fibroblasts, suggesting the risk score was positively correlated with the number of fibroblasts (Figure 4F).

### Clinical relevance of the risk signature and fibroblast content

**Figure 5:**
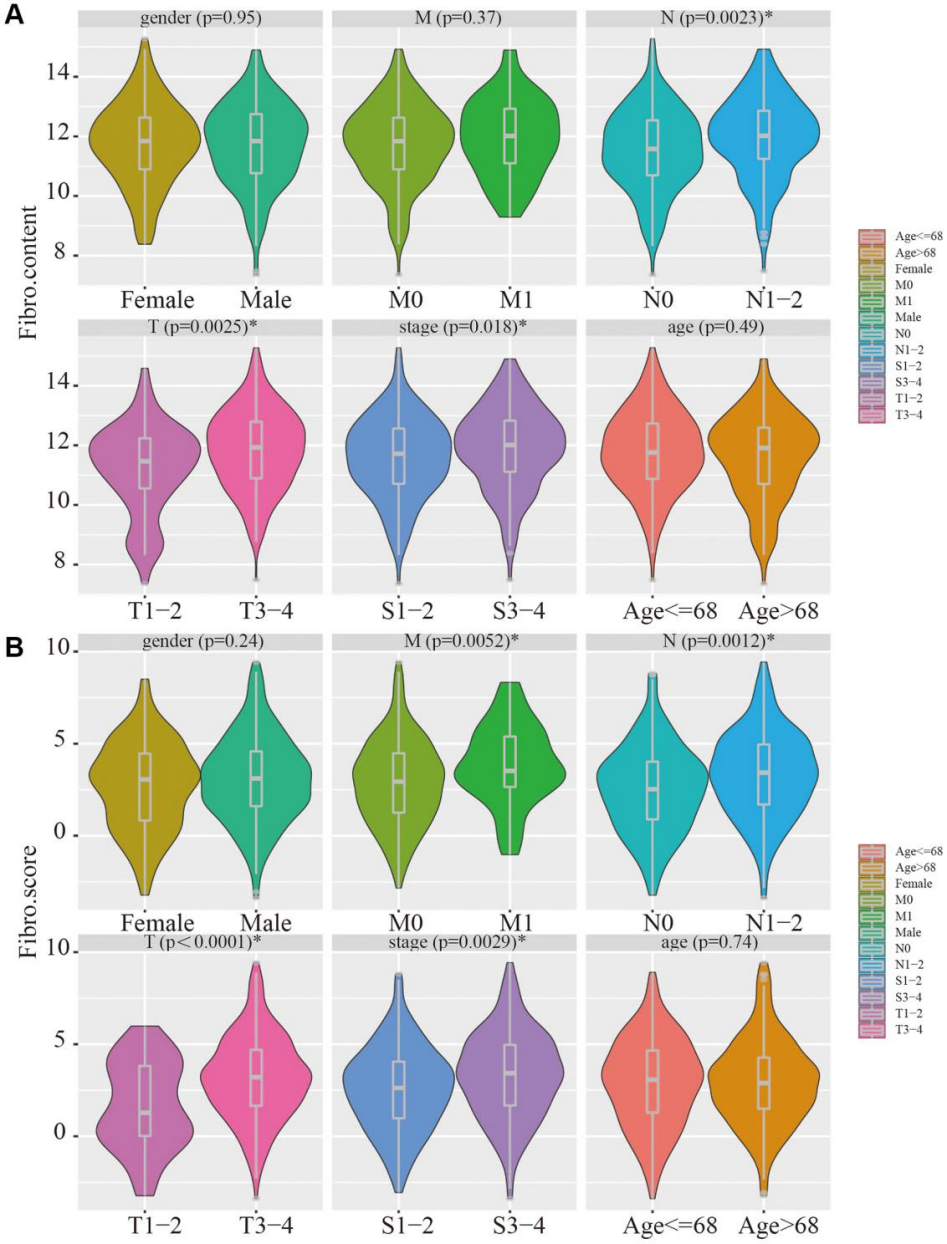
**Clinical relevance of fibroblast content and clinical relevance of risk score.** (**A**) The fibroblast content was significantly increased in the advanced stage, advanced T stage, and positive lymph node metastasis groups. (**B**) The risk score was significantly higher in the advanced stage, advanced T stage, positive lymph node metastasis, and distant metastasis groups.

The relevance of the fibroblast content relative to clinical traits including age, gender, stage, and TNM status was assessed using the TCGA database. The fibroblast content was significantly increased in the advanced stage, advanced T stage, and positive lymph node metastasis groups (Figure 5A). Assessment of the relationship between risk score and clinical traits in TCGA showed that the score was significantly higher in the advanced stage, advanced T stage, positive lymph node metastasis, and distant metastasis groups (Figure 5B).

### Mutation and copy number alteration analysis and protein-protein interactions of hub genes

**Figure 6:**
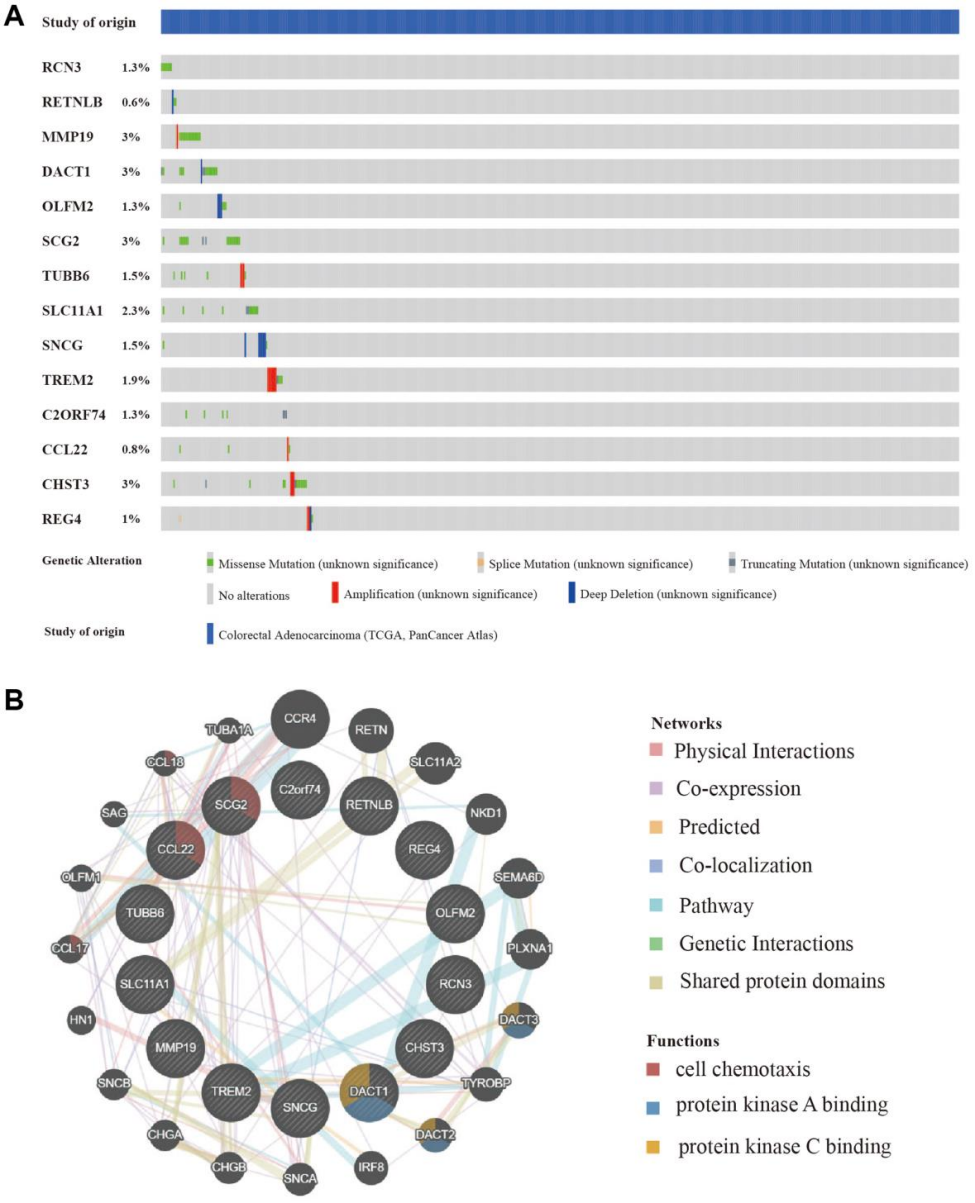
**Mutation and copy number alteration (CNA) analysis and protein-protein interactions (PPI) of hub genes.** (**A**) Mutation and copy number alteration of each hub gene. (**B**) The 20 functional similar genes were located in the outer circle, while hub genes were located in the inner circle. The color of nodes was related to the protein function while line color represented the type of protein interaction.

In addition to the analysis at the transcriptome level, we examined the role of marker genes at genome level. For this purpose, the cBioPortal was used to analyze the mutations of these genes in CRC. At the genome level, the mutation frequencies of the 14 genes were not significant; the main mutation types were missense mutations and amplifications. MMP19, DACT1, SCG2, and CHST3 were the most frequent CNAs with a 3% mutation rate among the 14 hub genes, whereas RETNLB was the least frequent CNA with a 0.6% mutation rate (Figure 6A).

To predict functionally similar hub genes, we used GeneMANIA to obtain the 20 most similar hub genes. The hub genes were located in the inner circle and the predicted genes were in the outer circle. The results showed that their functions were mostly related to cell chemotaxis and protein kinase binding which is essential for tumor progression and metastasis (Figure 6B).

### Molecular characteristics and pathways of the fibroblast-related risk signature

**Figure 7:**
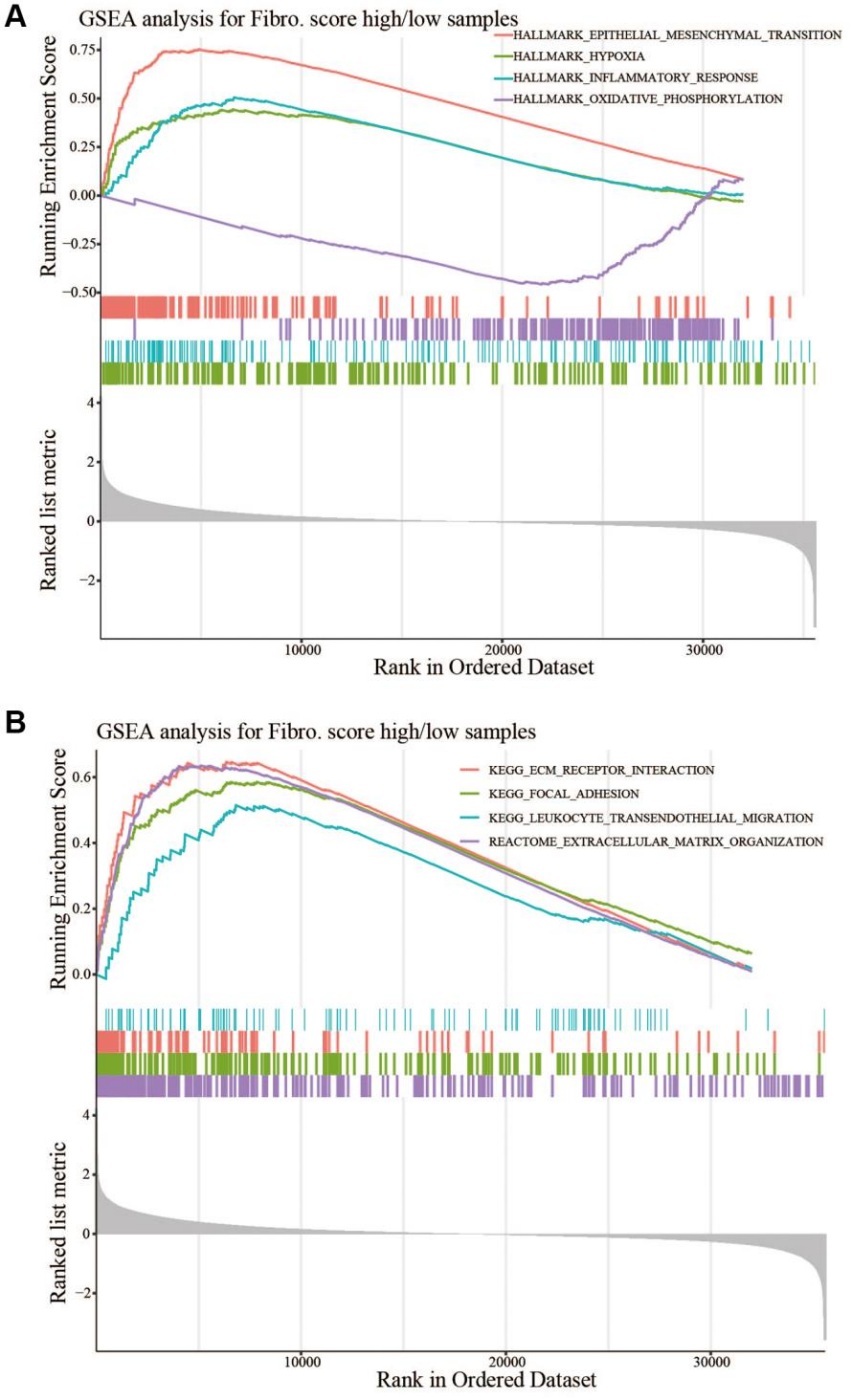
**GSEA between the high- and low-risk groups.** (**A**) HALLMARK_EPITHELIAL _MESENCHYMAL_TRANSITION, HALLMARK_HYPOXIA, and HALLMARK_INFLAMMATORY_RESPONSE were significantly upregulated in the high-risk group, and HALLMARK_OXIDATIVE_PHOSPHORYLATION was significantly upregulated in the low-risk group. (**B**) KEGG_ECM_RECEPTOR_INTERACTION, KEGG_FOCAL_ADHESION, KEGG_LEUKOCYTE_TRANSENDOTHELIAL_MIGRATION, and REACTOME_EXTRACELLULAR_MATRIX_ORGANIZATION were significantly upregulated in the high-risk group.

**Figure 8:**
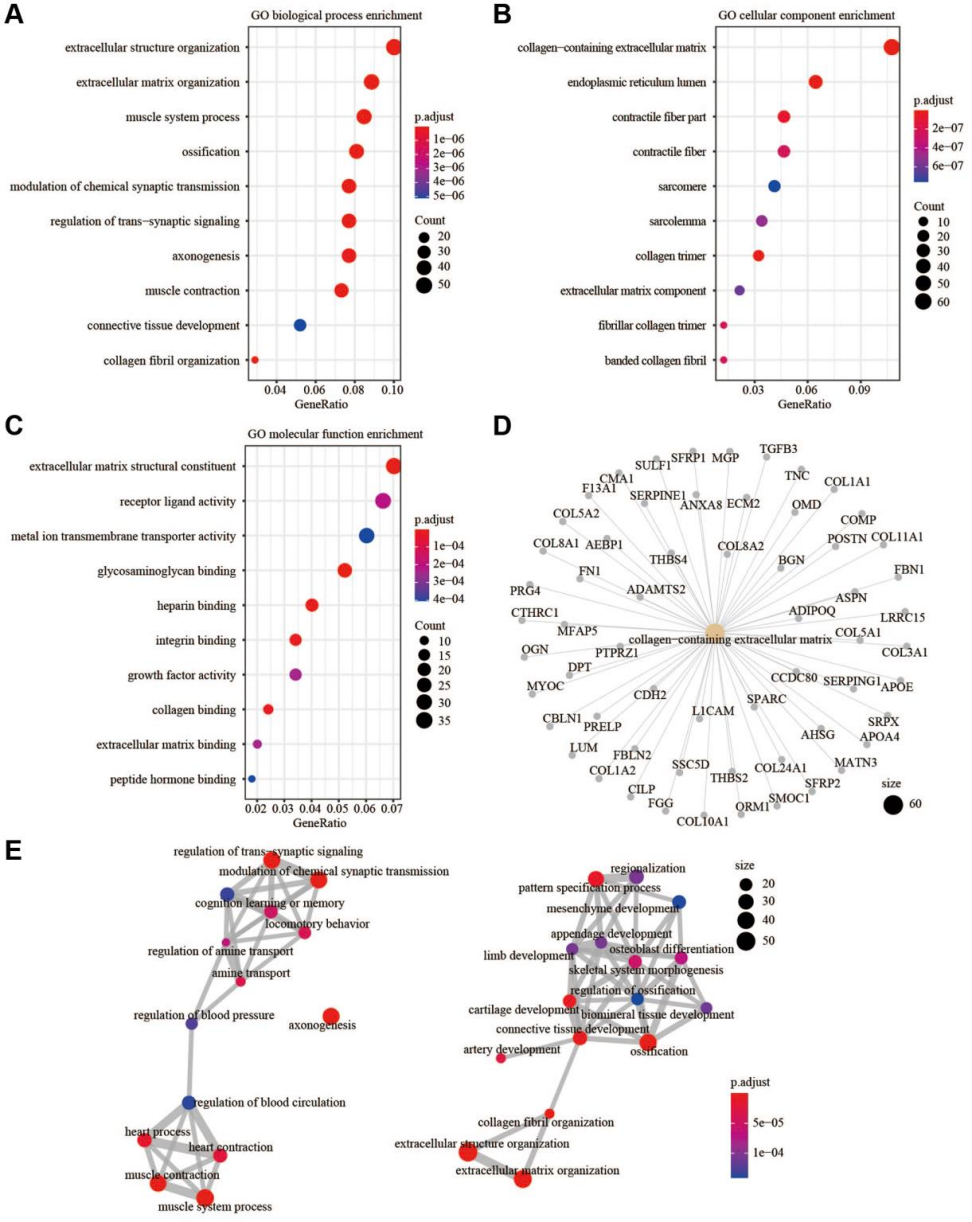
**GO enrichment analysis.** (**A**) GO biological process enrichment. (**B**) GO cellular component enrichment. (**C**) GO molecular function enrichment. (**D**) The up-regulated DEGs were related to collagen-containing extracellular matrix. (**E**) The relationship of different enriched biological processes.

Gene Set Enrichment Analysis (GSEA) of hallmark gene sets and pathway gene sets in the low-risk and high-risk groups from the training cohort was performed, and the four most enriched characteristics and pathways were selected. For the hallmark gene sets, HALLMARK_EPITHELIAL_MESENCHYMAL_TRANSITION, HALLMARK_HYPOXIA, and HALLMARK_INFLAMMATORY_RESPONSE were significantly upregulated in the high-risk group, and HALLMARK_OXIDATIVE_PHOSPHORYLATION was significantly upregulated in the low-risk group (Figure 7A). This suggested that the high-risk group has a higher progression potential and is characterized by inflammation and hypoxia. For pathway gene sets, KEGG_ECM_RECEPTOR_INTERACTION, KEGG_FOCAL_ADHESION, KEGG_LEUKOCYTE_TRANSENDOTHELIAL_MIGRATION, and REACTOME_EXTRACELLULAR_MATRIX_ORGANIZATION were significantly upregulated in the high-risk group (Figure 7B). Gene ontology (GO) biological process enrichment, GO cellular component enrichment, and GO molecular function enrichment were performed in the TCGA cohort. Functions related to the ECM were most enriched (Figure 8A–8C). For GO cellular component enrichment, some of the upregulated DEGs in high-risk group were related to the most enriched collagen-containing ECM (Figure 8D). For GO biological process enrichment, the relationship of different enriched biological processes was also established (Figure 8E).

### Correlations with immune cells and immune responses

**Figure 9:**
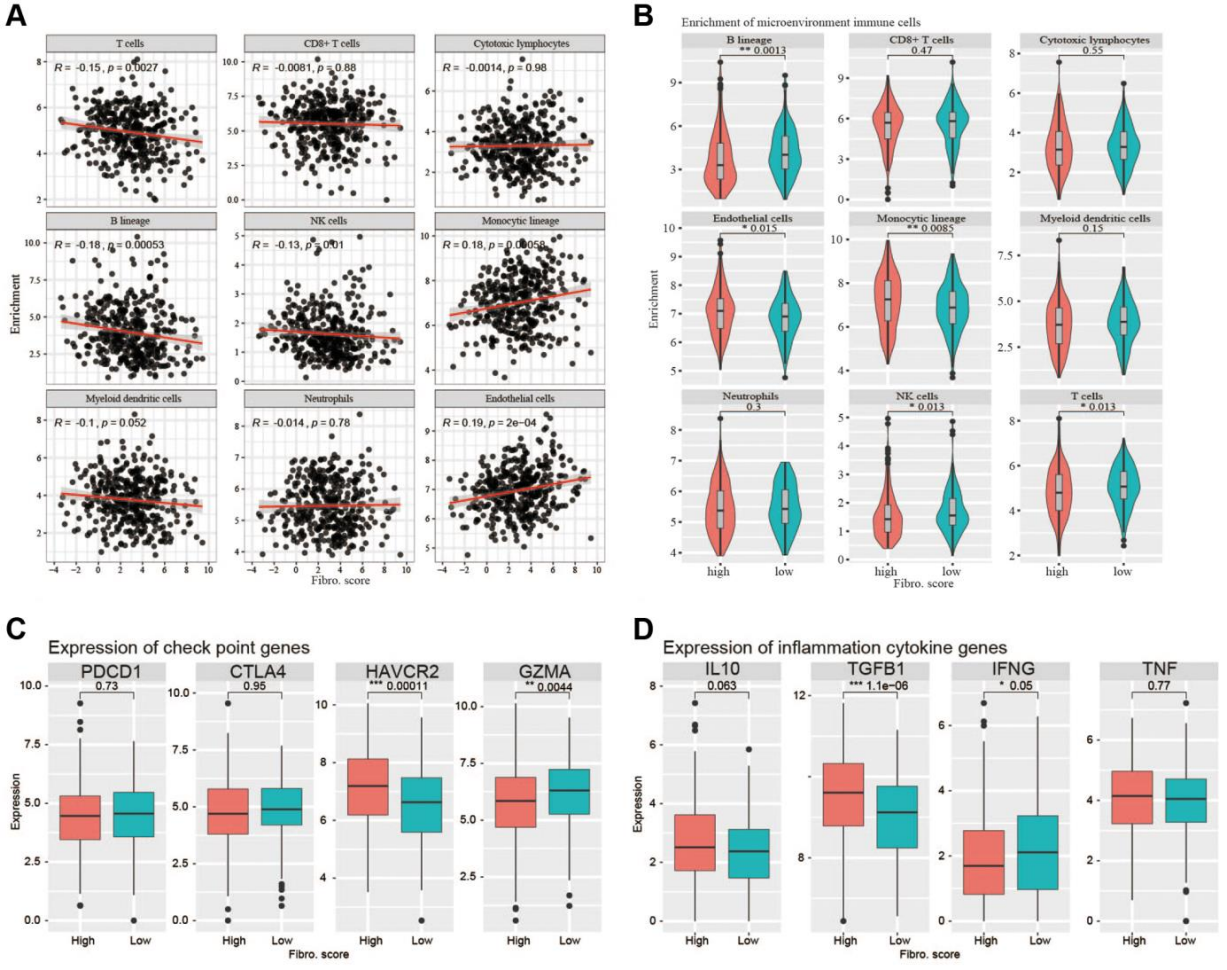
**Correlations with immune cells and immune responses.** (**A**) The B lineage cells, T cells, and NK cells were negatively correlated with the risk score, whereas endothelial cells and monocytic lineage content were positively correlated with the risk score. (**B**) The main immune cell lineages including B lineage, T cells and NK cells are higher in low-risk group (*P* < 0.05) while endothelial cells and monocytic lineage are higher in high-risk group. (**C**) The immune checkpoint genes (*PDCD1, CTLA4, HAVCR2, GZMA*) expression between high-risk group and low-risk group. (**D**) The inflammation cytokine genes (*IL10, TGFβ1, IFNG, TNF*) expression between high-risk group and low-risk group.

To determine whether the signature indicated the immune cell infiltration in the TME of CRC patients, we used MCP-counter to analyze the TME. The results showed that B lineage cells, T cells, and NK cells were negatively correlated with the risk score, whereas endothelial cells and monocytic lineage content were positively correlated with the risk score (Figure 9A). In the high-risk and low-risk groups, main immune cell lineages that suppress tumors including B lineage cells, T cells, and NK cells were higher in the low-risk group (*P* < 0.05), whereas endothelial and monocytic lineage cells were higher in the high-risk group (*P* < 0.05) (Figure 9B). EPIC was also used to confirm the correlation between TME and risk score, the result showed a higher correlation between TME and risk score (Supplementary Figure 3). CD8+T cells are important anti-tumor immune cells, and the content of CD8+T cells did not differ significantly between the two groups. Analysis of common immune checkpoint genes showed high expression levels of *HAVCR2* in the high-risk group, whereas *GZMA* was expressed at high levels in the low-risk group. This suggested that the content of exhausted CD8+T cells was higher in the high-risk group (Figure 9C). Analysis of inflammatory cytokine genes showed that the high-risk group had high expression of *TGFβ1*, whereas the low-risk group had high expression of *TNF*, implying an immunosuppressive TME in the high-risk group (Figure 9D).

## DISCUSSION

The CRC is one of the most commonly diagnosed and fatal cancers worldwide [1]. Changes in diet habits and aging may lead to an increase in the incidence of CRC [21].

Fibroblasts are stromal cells involved in many biological processes, including deposition of the ECM, regulation of epithelial differentiation, regulation of inflammation, and wound healing [4, 22, 23]. Previous genetic and cell biology studies also indicated that fibroblasts were involved in tumor growth. A subset of fibroblasts called cancer-associated fibroblasts (CAFs) are associated with cancer cells during the different stages of tumor progression [24]. At the initial stages of malignancy, fibroblasts could secrete TGFβ and hepatocyte growth factor to induce the initiation of cancer within the normal human epithelium [25]. Stromal cell-derived factor 1 and high-mobility group box 1 released by CAFs also contributed to cancer proliferation and stemness [26]. In addition, CAFs might enhance cancer cell invasion by affecting ECM stiffness [27]. During tumor metastasis, TGFβ1-stimulated CAFs secreted IL-11 to enhance the survival of CRC cells and increased the efficiency of organ colonization [28]. PDGF-stimulated CAFs enhanced CRC cell intravasation and promoted the formation of distant metastases via the secretion of stanniocalcin 1 [29]. Overall, fibroblasts did have a positive effect on tumor progression.

Based on TCGA and GEO, many risk models have been developed to predict the OS of CRC patients; most of these are mechanism-driven models based on factors such as aging, hypoxia, and autophagy [9, 10, 11]. However, a fibroblast-related risk signature based on open database has not been established to date.

In this study, we established a 14-gene risk signature, which was significantly associated with the OS of CRC patients. Subgroup analysis and independent prognostic analysis of univariate and multivariate COX demonstrated that our signature was stable in predicting the prognosis. The signature could assist physicians to perform individualized survival predictions, which would facilitate the selection of better treatment options.

In this study, PPI showed the function of hub genes focused on cell chemotaxis and protein kinase binding. In the CNA analysis, MMP19, DACT1, SCG2, and CHST3 were the most frequent CNAs, with a 3% mutation rate among the 14 hub genes. The enrichment analysis showed that EMT and ECM related pathways or functions that contribute to tumor invasion were enriched in the high-risk group. The analysis of TME showed more anti-tumor immune cells and less immunosuppressive environment in the low-risk group. Taken together, the risk signature could predict the OS of CRC patients and might involve multiple mechanisms.

Previous studies suggested fibroblasts in the TME expressed chemokines to attract and retain suppressive immune cells such as myeloid-derived suppressor cells, mesenchymal stem cells, and CD4^+^CD25^+^FOXP3^+^ regulatory T cells, which could counteract the anti-tumor functions of natural killer (NK) and CD8+ T and favor tumor progression [30–32]. Other chemokines secreted by fibroblasts could attract macrophages, neutrophils, and T cells toward the juxtatumoral stroma instead toward cancer cell nests [33, 34]. In addition to recruiting immunosuppressive cell types to the TME and deviating anti-tumor immune cell types from cancer nests, chemokines secreted by fibroblasts, such as CXCL8, CXCL12, and CCL2, had been implicated in polarizing resident macrophages and neutrophils toward a protumor versus an antitumor phenotype [35, 36]. Related biological process of fibroblasts might also involved in promoting tumor progression. Deposition of the ECM was an important function of fibroblasts; however, fibroblasts were also an important source of ECM-degrading proteases, which highlighted their role in maintaining ECM homeostasis [37, 38]. CAFs played a role in the invasion of cancer cells by pulling and stretching the ECM, resulting in the formation of small holes through which cancer cells could spread. As the main component of the ECM, collagens secreted by CAFs could also modulate crucial steps such as proliferation, apoptosis, angiogenesis, invasion, and metastasis to promote tumorgenesis [39]. The enhancement of EMT might also involved in progression of tumor, CAFs could secrete TGFβ1 to promote EMT, and then promoted tumor invasion and metastasis [40, 41]. Above all, the poor prognosis of patients in high-risk group might relate to the immune cell infiltration in the TME, EMT, and ECM related processes.

Our study had some limitation. First, many fibroblast marker genes in the testing group GSE39582 were default, so we had to use the DEGs between high-fibroblast and low-fibroblast groups to establish the signature. Second, this signature needed further experimental trials and large-scale clinical trials to validate.

In summary, we developed a fibroblast-related signature that could be applied as a novel prognostic assessment tool to predict the prognosis of CRC and further analysis of the 14 hub genes was necessary to explore their possible clinical value.

## CONCLUSION

We proposed a novel and efficient fibroblast-related risk signature by using accessible sequencing data of CRC patients. This would help to guide the disease management and individualized treatment of CRC patients.

## Supplementary Materials

Supplementary Figures
