# Supplementary material for: A prognostic fibroblast-related risk signature in colorectal cancer
Source: Aging (Albany NY). 2021 Nov 4;13(21):24251–70. doi: 10.18632/aging.203677 (PMC8610139; doi:10.18632/aging.203677)
Supplement: Supplementary Figures [file aging-13-203677-s001.pdf]

SUPPLEMENTARY FIGURES

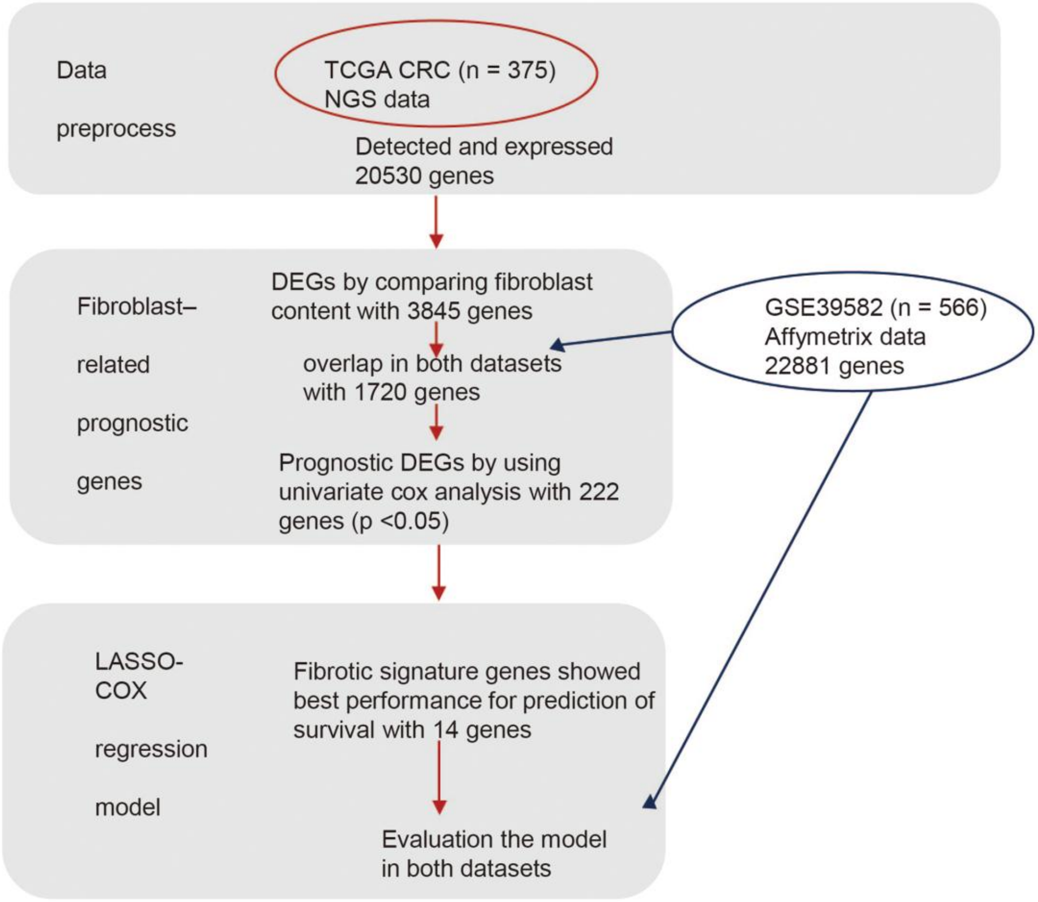

Supplementary Figure 1. The flow chart of the signature building process.

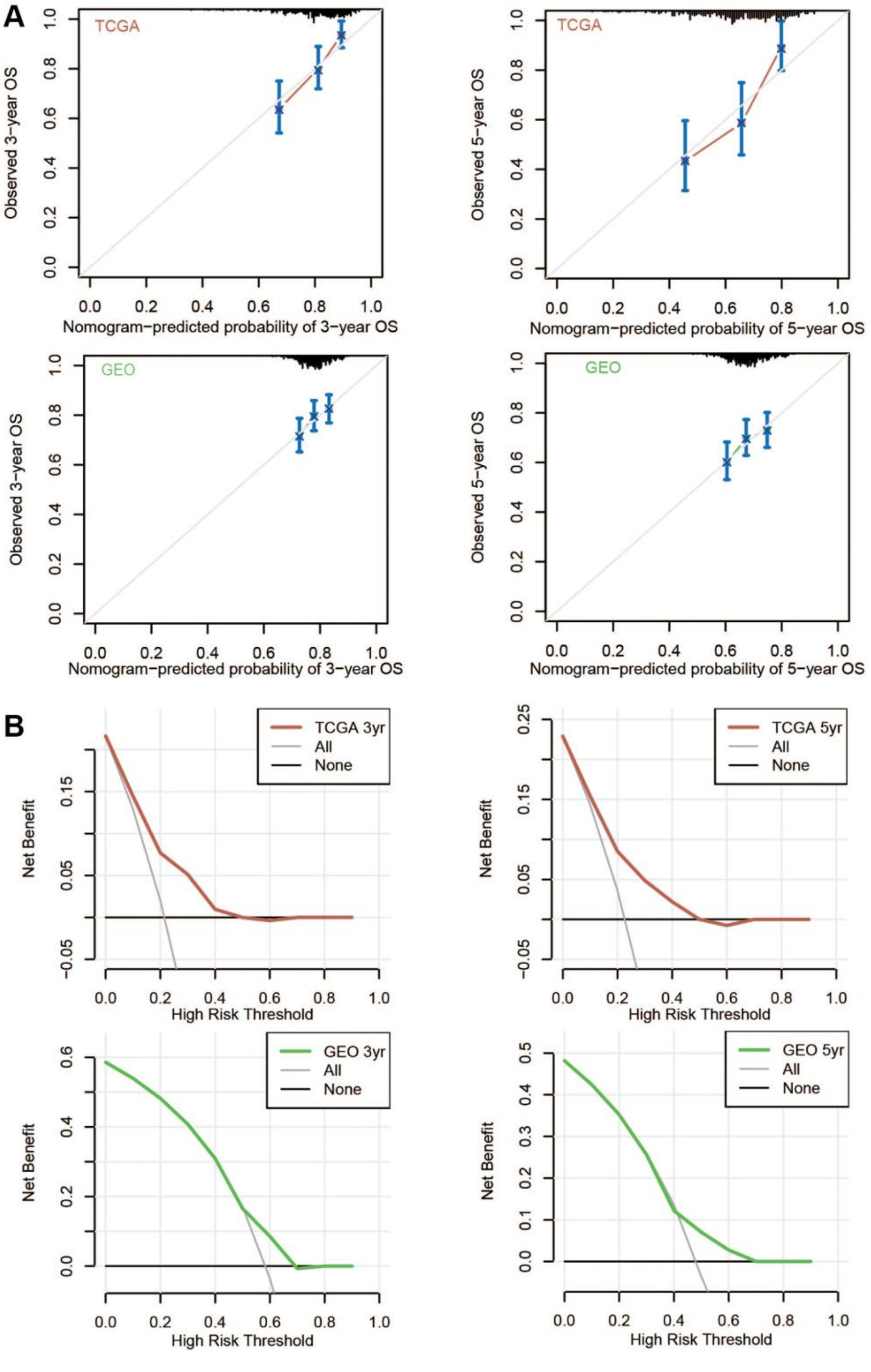

Supplementary Figure 2. The predictive performance of the prediction models. (**A**) Calibration curve for the prediction of 3-and 5-year overall survival. (**B**) DCA curve for the prediction of 3-and 5-year overall survival.

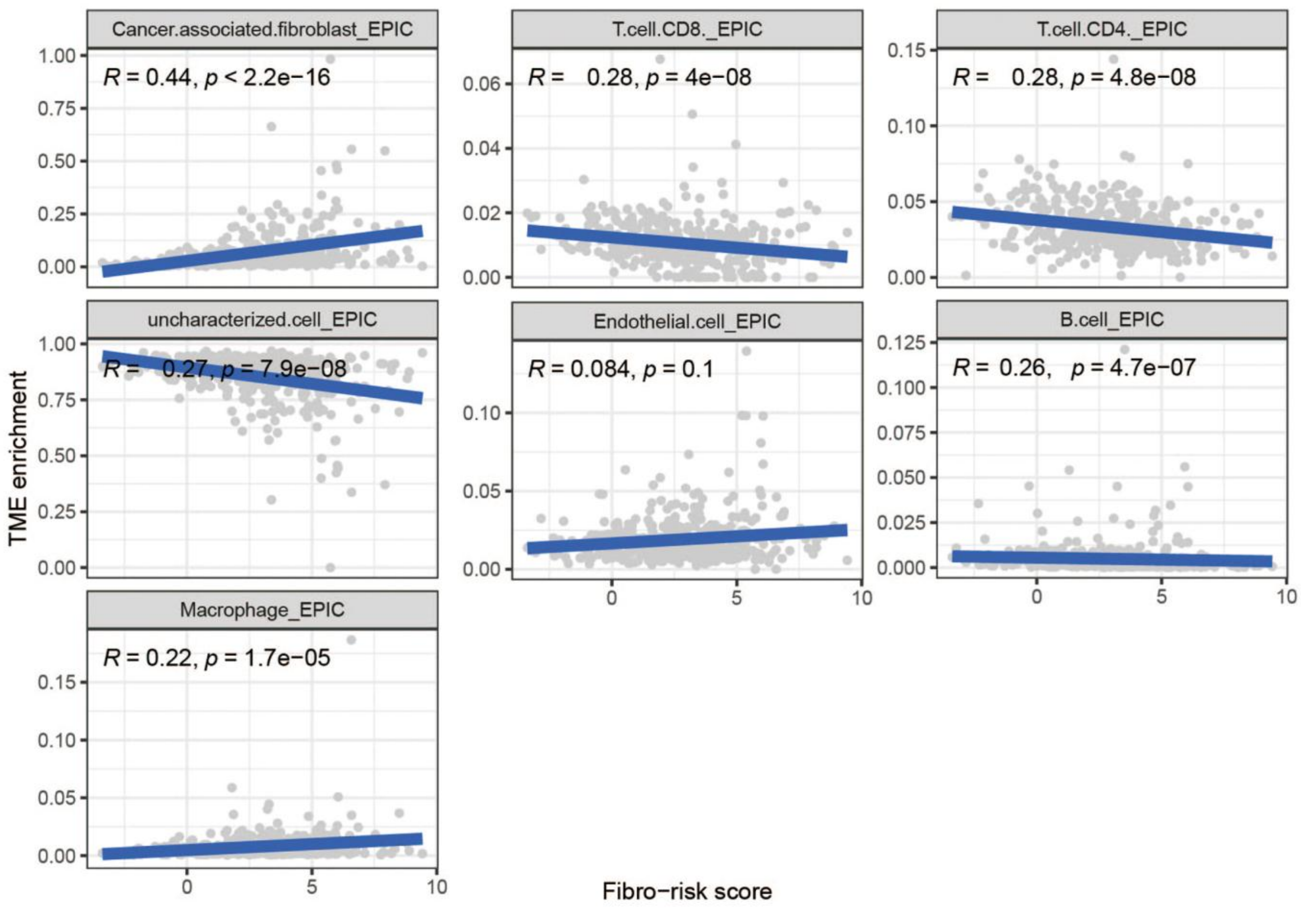

Supplementary Figure 3. Correlation between TME and risk score. The cells in TME have a higher correlation with the risk score.
